# Supplementary material for: Diversity of Leaf Fungal Endophytes from Two Coffea arabica Varieties and Antagonism towards Coffee Leaf Rust
Source: Plants (Basel). 2024 Mar 12;13(6):814. doi: 10.3390/plants13060814 (PMC11154406; doi:10.3390/plants13060814)
Supplement: Supplementary file 1 [file plants-13-00814-s001.zip › plants-2866745-supplementary.pdf]

Diversity of Leaf Fungal Endophytes from Two *Coffea arabica* Varieties and Antagonism towards Coffee Leaf Rust

Ruth A. Poma-Angamarca [1], Jacqueline R. Rojas [1], Aminael Sánchez-Rodríguez [1,†] and Mario X. Ruiz-González [1,2,*,†,‡]

[1] Departamento de Ciencias Biológicas y Agropecuarias, Universidad Técnica Particular de Loja, San Cayetano Alto s/n, Loja 1101608, Ecuador; ruthalex2003@yahoo.es (R.A.P.-A.); jerojasx@utpl.edu.ec (J.R.R.); asanchez2@utpl.edu.ec (A.S.-R.)
[2] SENESCYT is the Secretaría de Educación Superior, Ciencia, Tecnología e Innovación from the government of Ecuador.Proyecto Prometeo SENESCYT, Universidad Técnica Particular de Loja, San Cayetano Alto s/n, Loja 1101608, Ecuador
* Correspondence: maruigon@upvnet.upv.es
† Joint Senior Authors.
‡ Current address: Institute for Conservation and Improvement of Valencian Agrodiversity, Universitat Politècnica de València, Camino de Vera s/n, 46022 Valencia, Spain.

Supplementary Table S1. Identification of fungal endophytes in Caturra and Colombia coffee varieties. The taxonomic group was assessed for the samples isolated in this work (NCBI GenBank IDs in brackets, otherwise, determined after morphological resemblance) after blastn similarity with the GenBank ID of the closest taxa (percent identity in bold). The Days column shows the number of days before the endophyte detection in MYG Agar plates. The Ecology column describes the ecology (and geographic origin) of the closest taxid, with bibliographic references when available.

Taxonomic group	Sample ID (GenBank ID)	Closest taxa (GenBank ID: % ID)	Ecology (Origin)	Ref.	Days
**ASCOMYCOTA**					
**Dothideomycetes**					
**Incertae sedis**					
Fungal endophyte sp. 1	F112 (MK005037)	EU687041: **90.22**	Endophyte from *Rhipidocladum racemiflorum* (Panama)	1	12
**Capnodiales; Cladosporiaceae**					
Uncultured Cladosporiaceae	F129**	GQ517141: **86.88**	Endophyte from *Quercus macrocarpa* (USA)	2	4
**Pleosporales; Sporormiaceae**					
Preussia pseudominima	F041 (MK005019)	KU204603: **99.24**	Endophyte from *Daphnopsis americana* (Costa Rica)	3	4
Preussia sp.	F134 (MK005045)	KR093940: **95.20**	In fungus garden of *Atta sexdens rubropilosa* (Brazil)	4	4
**Eurotiomycetes**					
**Eurotiales; Aspergillaceae**					
Penicillium cf. citrinum	F096, F126 (MK005040)	KF498874 / MN788117 /	Endophyte from *Pogostemon cablin* / mould	5	3-4

		MN788106: **91.12**	associated with Cocoa (China / Nigeria)		
**Trichocomaceae**					
Talaromyces cf. oumae-annae	F022 (MK005060), F081, F099	OM791640 / MH934969 / MT530189: **89.88**	Endophyte from *Arachis hypogaea* / entomogenous fungi (India / Malaysia)		3-8
**Sordariomycetes**					
Fungal endophyte sp. 2	F065 (MK005023)	KT289540: **96.19**	Endolichenic fungus (Costa Rica)	6	6
**Diaporthales; Diaporthaceae**					
Diaporthe australafricana	F118 (MK005038)	MF280391 / OM975589: **95.34**	Phytopathogen (Chile) / Endophyte (USA)		6
**Glomerellales; Glomerellaceae**					
Colletotrichum acutatum complex					
C. aenigma	F026, F132 (MK005044)	ON329227: **97.95**	Anthracnose (Asia)	7	6
C. cf. fioriniae	F113 (MK005058)	ON368204: **89.00**	Bitter Rot Disease (USA)	8	4
C. lupini	F072 (MK005027), F073, F075, F082	MH178095: **99.14**	Isolated from *Lupinus micranthus* Guss (China)		4-6
C. scovillei	F043 (MK017761), F045, F057	LC488868: **99.15**	Anthracnose (Viet Nam)		4
Colletotrichum sp. 1	F042 (MK005049), F058	MK005027 (F072): 91.53 MK005048 (F040) / MH865411 / MH854629: **91.45**	(Colombia)	9	4
Colletotrichum sp. 2	F036, F039, F040 (MK005048), F046, F102	EF687919: **92.65**	Endophyte from *Coffea arabica* Colombia (Colombia)	10	4
Colletotrichum sp. 3	F127 (MK005055)	JQ894656: **91.74**	Isolated from *Mangifera indica* (India)		4
C. boninense species complex					
C. boninense / *C. cymbidicola*	F066 (MK005051), F080 (MK005052)	MK005052 (F080): 97.70 / MN458530: **97.30** / MT464454: 97.32	Endophyte from *Justicia brandegeeana* (Brazil)	12	4
Colletotrichum sp. 4	F011* (MK005046), F078, F084, F133	OL842171: **90.40**	Endophyte of aquatic plants, China	11	3-4
C. gloeosporioides species complex					

C. karstii	F059 (MK005022), F083 (MK005031), F085 (MK005032), F050 (MK005020), F079 (MK005030), F077 (MK005029), F095 (MK005035), F031, F068, F089, F098, F100	OM436864: 96.95 / MN842791: 97.66 / OP445269: 98.14 / KX578788: **98.80** / KX578788: 98.80 / OM436864: 98.51 / OP782678: 93.77 / KX578788 / MK005029: 93.52	Endophyte / *Citrus limon* (South Africa)	13	4-7
C. musae	F007* (MK005009), F029	OW988162: **94.84** / KX069828: 94.66	Anthracnose *Musa acuminata* (Australia)		4-5
Colletotrichum sp. 5	F053 (MK005050), F094	MK005055 (F127): **91.21** / OK030873: 89.36	Crown rot on strawberries (USA)		4-5
**Glomerellales; Plectosphaerellaceae**					
Musicillium theobromae	F017*, F019* (MK005015), F103	MK579179: **95.84**	Isolated from *Solanum betaceum* root (New Zeland)		5-10
**Hypocreales; Clavicipitaceae**					
Beauveria sp.	F117				7
**Sordariales**					
Fungal endophyte sp. 3	F104**	KF435260: **86.97**	Endophyte (Panama)	14	10
**Sordariales; Chaetomiaceae**					
Chaetomium sp.	F060, F074 (MK005028)	MF495440 / KF435950 / KF435726 / KF435552 / KF435385: 8**7.70**	Endophyte (Panama)	14	4-6
**Trichosphaeriales; Trichosphaeriaceae**					
Nigrospora cf. oryzae	F025 (MK005047), F030, F034**	MT123068: **91.94**	Endophyte (China)		5-10
Nigrospora sp. 1	F128 (MK005041)	MN341467: **89.74**	Endolichenic from *Peltigera neopolydactyla* (South Korea)	15	7
**Xylariales; Hypoxylaceae**					
Annulohypoxylon cf. *stygium*	F015 (MK005013)	KP133169: 98.75	Endophyte from *Miconia floribunda* (Ecuador)	16	6
Hypoxylon sp. 1	F071 (MK005026)	FJ612775: **96.97**	Endophyte from *Cecropia insignis* seeds (Panama)	17	11
**Xylariales; Xylariaceaea**					

Fungal endophyte sp. 4	F106 (MK005036)	EU687119: **97.13**	Endophyte (Panama)	1	4
Fungal endophyte sp. 5	F064**	KU747690 / FJ612923: **75.39**	Endophyte (Panama)	17, 19	4
Anthostomella sp.	F121 (MK005059)	JQ754021: **91.14**	Endophyte (Brazil)	20	6
Lopadostoma polynesium	F001*, F002* (MK005007)	KC774600: **91.11**	Isolated from bark of *Amelanchier ovalis*	21	7
Nemania difusa	F125 (MK005039)	KF435731: **97.88**; MF770851: 97.38	Endophyte from *Myconia* sp. leaves (Panama)	1	4
Xylaria adscendens	F086 (MK005033), F035 (MK005017), F032 (MK005016), F087 (MK005034), F097 (MK005054), F131 (MK005043), F047, F048, F055	KP133288: **96.92**, 95.79, 95.07, 95.60, 93.68, and 95.18	Endophyte from *Myconia brevitheca (*Ecuador)	16	4-5
Xylaria cf *berteroi*	F088 (MK005053), F012*, F051, F054, F124	MK334005 / MK247857: **98.75**	Disease of *Actinidia chinensis* cv. Hongyang (China)		3-5
Xylaria curta	F070 (MK005025)	KJ883611: **97.21**	Endophyte from *Coffea arabica* L. (Puerto Rico)		4
Xylaria multiplex	F009* (MK005011), F052	MN833802 / KT289626 / KP133438: **94.22**	Endophyte from leaves (Ecuador)	16	4-5
Xylaria sp. 1	F008* (MK005010), F021	KF467102: **94.59**	Endophyte from *Herrania nycterodendron* (Ecuador)		5-10
Xylaria sp. 2	F016* (MK005014), F114	MH003490: **96.34**	Endophyte from *Polylepis incana* (Ecuador)		6-12
Xylaria sp. 3	F067 (MK005024), F037 (MK005018), F010*, F063	KF435704: **98.54**, / MK005018 (F037) 97.24	Endophyte from *Myconia chrysophylla* (Panama)	14	4-6
Xylaria sp. 4	F023 (MK005056)	MH003401: **92.02**	Endophyte from *Polylepis lanuginosa* (Ecuador)		10
Xylaria sp. 5	F130 (MK005042)	MT992054: **94.33**	Endophyte from *Otoba gracilipes* (Colombia)		4
Xylaria sp. 6	F027**, F044, F056 (MK005021), F061, F062, F076, F101	JQ341084: **98.23**	Endophyte from *Diospyros crassiflora* (Cameroon)		4-6
**BASIDIOMYCOTA**					

**Agaricomycetes**				
**Hymenochaetales; Schizoporaceae**				
Xylodon sp.	F107**	OM891735: **87.3**	wood-rotting fungi (China)	5
**44 taxonomic groups**				

* Denotes samples that were identified in the pre-experiment, and were not included in the analyses.

** Denotes sequences of low quality.

References:

1. Higgins et al. *Mycologia* **2011**, 103, 247–260;

2. Jumpponen et al. *New Phytol* **2010**, 186, 496-513;

3. Rojas-Jimenez & Tamayo-Castillo. Fungal Endophytes and Their Bioactive Compounds in Tropical Forests of Costa Rica. In Neotropical Endophytic Fungi; Rosa, L. H. Ed.; Springer Nature Switzerland AG, **2021**; 109-130;

4. Pereira et al. *Fungal Ecol* **2016**, 21, 68-75;

5. Akinfala et al. *Int J Food Microbiol* **2020**, 316, 108490;

6. Arnold et al. *Syst Biol* **2009**, 58, 283-97;

7. https://www.cabidigitallibrary.org/doi/10.1079/cabicompendium.90045598 (27/12/2023);

8. Martin et al. *Phytopathology* **2021**, 111, 966-981;

9. Vu et al. *Stud Mycol* **2019**, 92, 135-154;

10. Vega et al. *Fungal Ecol* **2010**, 3, 122-138;

11. Zheng et al. *J Fungi* **2022**, 8, 87;

12. Silva et al. South *Afr J Bot* **2020**, 134, 359-368;

13. Douanla-Meli et al. *Fungal Biol* **2017**, 121, 858-868;

14. Higginbotham et al. *PLoS ONE* **2013**, 8, e73192;

15. Oh et al. *Sustainability* **2020**, 12, 3769;

16. Thomas et al. *Biotropica* **2016**, 48, 381–393;

17. U'Ren et al. *Mycol Res* **2009**, 113, 432–449;

18. Hsieh et al. *Mycologia* **2005**, 97, 844–865;

19. Del Olmo-Ruiz & Arnold. *J Trop Ecol* **2016**, 33, 60-73;

20. Gonzaga et al. *J Appl Microbiol* **2015**, 118, 485-96;

21. Jaklitsch et al. *Persoonia* **2014**, 32, 52-82.
